# Acute surgical site infection after total knee arthroplasty in patients with rheumatoid arthritis versus osteoarthritis

**DOI:** 10.1038/s41598-021-02153-x

**Published:** 2021-11-22

**Authors:** Ho-Ken Chung, Shu-Hui Wen, Wei-Chuan Chang, Kuan-Lin Liu

**Affiliations:** 1 Department of Orthopedics, Buddhist Tzu Chi General Hospital, Hualien, 970473 Taiwan; 2 Department of Public Health, College of Medicine, Tzu Chi University, Hualien, 970374 Taiwan; 3 Department of Medical Research, Buddhist Tzu Chi General Hospital, Hualien, 970473 Taiwan; 4 School of Medicine, Tzu Chi University, Hualien, 970374 Taiwan

**Keywords:** Outcomes research, Rheumatic diseases

## Abstract

Osteoarthritis is the main cause for total knee arthroplasty (TKA), followed by rheumatoid arthritis. Previous studies have reported conflicting results concerning the risk of surgical site infection after TKA for rheumatoid arthritis and osteoarthritis patients. Thus, this study aimed to examine whether rheumatoid arthritis patients had a higher risk of acute surgical site infection after TKA compared to osteoarthritis patients. We conducted a retrospective cohort study using Taiwan’s National Health Insurance Research Database of the whole population from 2012 to 2015, and collected the medical records of osteoarthritis patients or rheumatoid arthritis patients who underwent TKA. To evaluate the risk of acute surgical site infection in rheumatoid arthritis patients, propensity score matching was implemented for osteoarthritis patients. Acute surgical site infection was observed in 2.58% of TKA cases in rheumatoid arthritis patients and 2.66% of TKA cases in osteoarthritis patients. Rheumatoid arthritis and osteoarthritis patients had comparable risk for 90-day (odds ratio = 0.81, 95% confidence interval: 0.371–1.768) and 1-year (odds ratio = 0.463, 95% confidence interval: 0.121–1.766) surgical site infection. In conclusion, patients with rheumatoid arthritis were not at higher risk of acute surgical site infection after TKA compared to osteoarthritis patients. The current treatment strategy for patients with RA is safe and appropriate if they require TKA.

## Introduction

Total knee arthroplasty (TKA) is currently considered the most successful treatment for end-stage arthritis of the knee and has a low complication rate. The main cause of end-stage arthritis is osteoarthritis (OA), which accounts for 90% to 97% of the primary indication for TKA, followed by rheumatoid arthritis (RA)^1^. RA is the most common inflammatory arthritis type. Its incidence rate in Taiwan was 15.2 per 100,000 people in 2007^2^. The outcome of TKA in RA patients is similar to the outcome in OA patients^3^. However, surgical site infection is a major concern and influences long-term TKA survival. The rate of surgical site infection is < 2%^4^, and the rate reported in Taiwan was 0.6% in 2017^5^. This complication mostly occurs within 1 year after TKA. Pulido et al. reported that 27% of infections were diagnosed within 30 days, whereas 65% of infections occurred within 1 year after arthroplasty^6^. Postoperative infection may result in significant morbidity and mortality and may account for massive healthcare expenditure. Several risk factors contribute to such catastrophic consequences, including obesity, diabetes mellitus, wound drainage or dehiscence, blood transfusion, coagulopathy, malignancy, immunodepression status, or rheumatoid arthritis^7–9^.

It has been suggested that RA patients are more vulnerable to infections due to the consumption of immunosuppressive drugs^10,11^. Comparisons of the TKA surgical site infection rate between RA and OA patients have been an interesting topic in this area^12–14^. Regarding this, two nationwide population studies reported increased surgical site infection in RA patients compared to OA patients^15,16^. Lee et al. also concluded that RA patients had a significantly higher rate of deep surgical site infection than OA patients did, but their superficial infection rates were similar^17^. However, other studies reported no increase in infection rate in RA patients compared to OA patients^18,19^. Thus, this topic remains controversial and warrants further investigation. In light of the current evidence, our aim is to examine whether the acute surgical site infection rate after TKA in RA patients is higher than that in OA patients.

## Methods

### Data sources

We conducted a retrospective population-based cohort study using Taiwan’s National Health Insurance Research Database (NHIRD). The NHIRD contains comprehensive claims data, including outpatient visits, patient admissions, and drug prescription details for > 99% of Taiwan’s population. In this study, we identified study samples from the whole population of the NHIRD from 1 January 2012 to 31 December 2015. The NHIRD contains demographic characteristics, surgical procedures, and diagnostic codes in accordance with the International Classification of Diseases, Ninth Revision, Clinical Modification (ICD-9-CM). The validity of ICD-9-CM diagnosis is reliable in several diseases^20^. We also retrieved basic information on contracted medical facilities from the NHIRD. For research purposes, these datasets can be linked by the patient’s or hospital’s scrambled identification number.

This study was conducted in accordance with the Declaration of Helsinki and was approved by the institutional review board at our hospital. The approved period of investigation was from January 2012 to December 2015. The requirement for obtaining informed consent from patients was waived due to the retrospective nature of the study (Research Ethics Committee in Hualien Tzu Chi Hospital, Buddhist Tzu Chi Medical Foundation; REC No.: IRB107-211-C).

### Study samples

**Figure 1:**
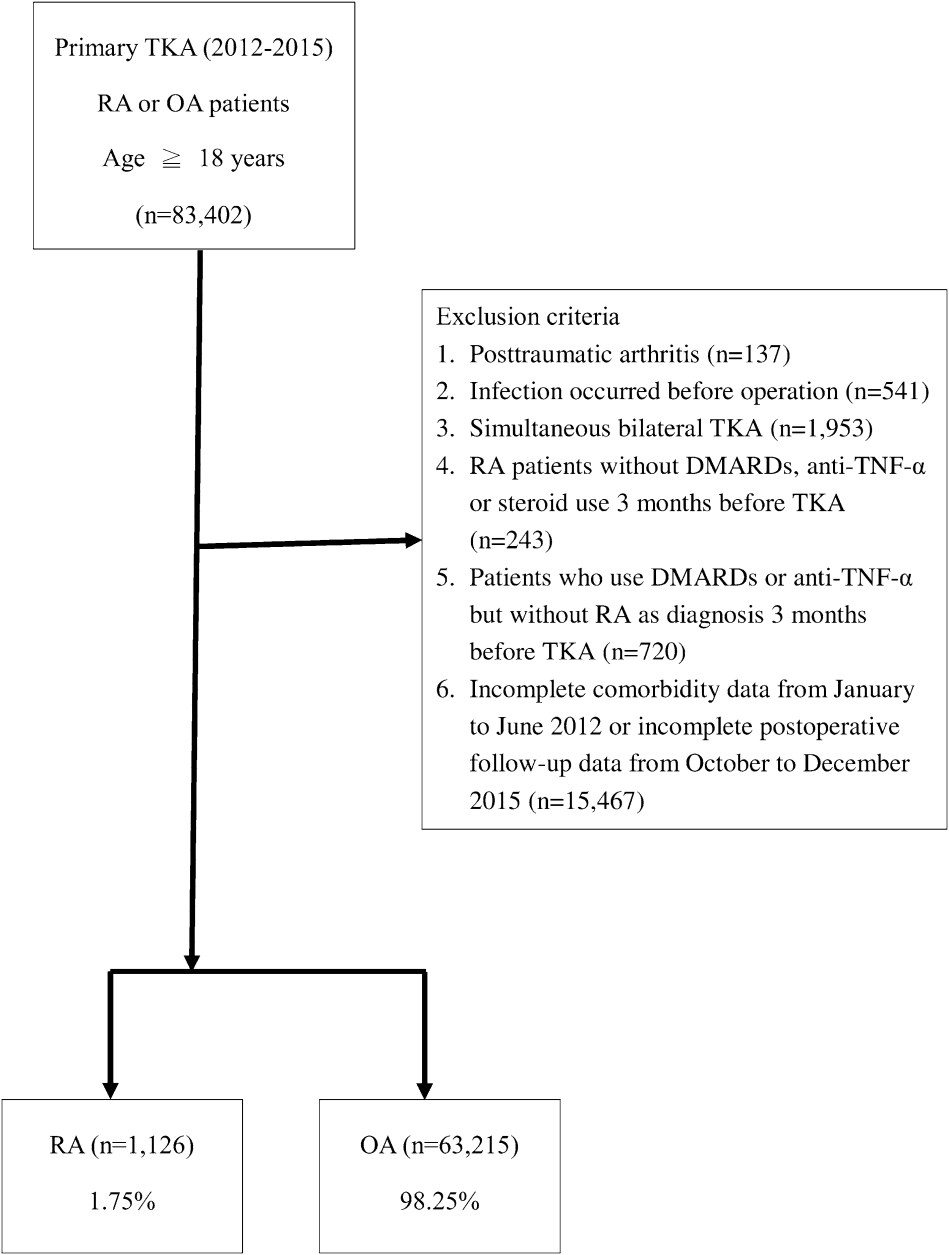
Flow chart of patient inclusion. DMARDs, disease-modifying antirheumatic drugs; OA, osteoarthritis; RA, rheumatoid arthritis; TKA, total knee arthroplasty; TNF, tumor necrosis factor.

We enrolled adult OA and RA patients who underwent primary TKA between 2012 and 2015. The sample size was calculated using Epitools (http://epitools.ausvet.com.au). For a cohort study with preassumed 5% of infection rate in OA patients, relative risk of 1.6^16^, 95% confidence level, and 80% power, the minimum sample size was estimated as 1057 per group. OA was identified by ICD-9-CM code 715.0 and RA by code 714.0. TKA was identified by ICD-9-CM procedure code 81.54 (Appendix A). Patients with post-traumatic arthritis or receiving simultaneous bilateral TKA were excluded (Fig. 1). Kim et al. suggested using both diagnosis code and disease-modifying anti-rheumatic drug (DMARD) prescriptions to identify RA patients in order to reach a high positive predictive value (86.2–88.9%) in databases^21^. Based on this suggestion, we used an algorithm for validation of the RA diagnosis. When RA diagnosis by ICD-9-CM code 714.0 from inpatient records in the NHIRD was viewed as the reference, the positive predictive value (PPV) was 82% for the algorithm using our criteria: ICD-9-CM code 714.0 from inpatient records along with ever-used pre-operative DMARDs, anti-tumor necrosis factor-α (anti-TNF-α), or steroids. Thus, to enhance the accuracy of identifying RA patients, we collected medication information including nonsteroidal anti-inflammatory drugs, DMARDs, anti-TNF-α, and steroids according to the Anatomical Therapeutic Chemical code. For example, methotrexate as a DMARD was labeled as L01BA01; etanercept as an anti-TNF-α product was labeled as L04AB01, and methylprednisolone as a steroid was labeled as H02AB04. We ruled out RA patients not receiving pre-operative DMARDs, anti-TNF-α, or steroid treatment, as well as patients treated with pre-operative DMARDs or anti-TNF-α but without an RA diagnosis (Fig. 1).

### Outcomes and covariates

The primary outcome was acute surgical site infection. Yokoe et al. concluded that nearly 60% of surgical site infections occurred within 30 days following surgery, and 74% of surgical site infections occurred within 90 days^22^. Considering this evidence, we defined acute surgical site infection as an infection that occurred within 90 days postoperatively, which covers three-quarters of surgical site infection, as this could appropriately reflect the risk related to surgery. Infection was recognized by diagnostic codes or procedural codes. Diagnostic codes include ICD-9-CM codes for intra-articular infection (996.60, 996.66, 996.67, and 996.69), postoperative infection (998.3 and 998.59), and pyogenic arthritis and osteomyelitis (711.06, 711.08, 730.26, and 730.28) following TKA. Procedural codes include the operation performed for the infection (48004C, 48005C, 48006C, 64004C, 64053B, and 64198B) (Appendix A). These two methods were also suggested by the program of healthcare-associated infection indicators conducted by the Taiwan Centers of Disease Control^23^. Patients either with infection occurring before TKA or not able to be followed up for 90 days were excluded (Fig. 1).

Patient covariates included demographic factors (age and sex), comorbidities, and surgery-related factors. Comorbidities were hypertension, diabetes mellitus, hyperlipidemia, and other diseases that might be associated with surgical site infection. These diseases were determined at baseline, 6 months prior to TKA and were identified by ICD-9-CM codes, including hypertension (401–405), diabetes mellitus (250), hyperlipidemia (272), chronic obstructive pulmonary disease (490–496), urinary tract infection (599.0), congestive heart failure (428), cancer (140–239), chronic renal disease (585), peripheral vascular disease (443.9), anemia (280–285), valvular heart disease (394–396, 424, and 746), and ischemic heart disease (410–414). Patients without complete comorbidity data were excluded (Fig. 1). Surgery-related factors included blood transfusion, which was identified by order codes 93001C, 93002C, and 93019C (Appendix A); operation year; length of stay; and hospital level.

### Statistical analysis

To minimize the possible selection bias caused by differences in baseline characteristics between the RA and OA patients, we adopted propensity-score (PS) matching based on the theoretical model for estimates of PS in the logistic regression model. Under PS matching, RA patients were matched with OA patients in a 1:3 ratio. PS was calculated using logistic regression with covariates, including sex, age, comorbidities, and operation year. RA and OA patients were matched based on greedy matching with caliper set at 0.2 times the standard deviation of PS. The chi-squared test, Fisher’s exact test, and an independent sample t-test were utilized for the comparison of characteristics of the RA and OA patients before and after PS matching. The standardized mean difference (SMD) was calculated after matching, in which SMD < 10% indicates appropriate matching. *p-*value < 0.001 was considered statistically significant.

We used a multivariable regression analysis to compare perioperative infection risk between RA and OA patients. After matching, unmatched OA patients were ruled out. We performed two analyses to estimate the acute infection risk of RA patients after TKA—namely, PS weighting with standardized mortality ratio weighting (SMRW) and conditional logistic regression for PS-matched data. According to SMRW, the weight for the RA group was 1, while the weight for the OA group was the ratio of PS and 1-PS. Two corrected methods were arranged: Model 1—correction with diagnosis (RA/OA); Model 2—correction with diagnosis (RA/OA), sex, age, hypertension, hyperlipidemia, diabetes mellitus, length of stay, blood transfusion, and hospital level. Other comorbidities were not included in the correction because of a low sample size, making their data not usable in logistic regression.

Moreover, a sensitivity analysis was conducted by repeating the above analyses to observe the risk of surgical site infection using data obtained 1 year after TKA. We ruled out incomplete data between January and December of 2015. All statistical analyses were performed with SAS version 9.4 statistics software (SAS Institute, Inc., Cary, NC, USA).

### Ethics declarations

This study was conducted according to the guidelines of the Declaration of Helsinki, and approved by the Institutional Review Board at Hualien Tzu Chi Hospital (Research Ethics Committee in Hualien Tzu Chi Hospital, Buddhist Tzu Chi Medical Foundation; REC No.: IRB107-211-C, October/22/2018).

### Informed consent statement

Patient consent was waived due to the retrospective nature of the study.

## Results

A total of 64,341 patients who had undergone TKA between July 2012 and September 2015 were involved in this study, of whom 1.8% (n = 1126) were diagnosed with RA. The characteristics of the population are listed in Table 1.

**Table 1:** Characteristics of the study population at baseline and 90-day follow-up after TKA.

		Original data				After 1:3 PS matching			
		RA (n = 1126)	OA (n = 63,215)	*p*-value	SMD	RA (n = 1066)	OA (n = 3198)	*p*-value	SMD
PS		0.05 ± 0.06	0.02 ± 0.02	< 0.001	0.671	0.03 ± 0.03	0.03 ± 0.03	0.954	< 0.001
Age		64.76 ± 10.24	70.87 ± 8.2	< 0.001	0.689	65.71 ± 9.36	65.99 ± 9.3	0.389	0.03
Sex	Male	186 (16.52)	16,431 (26.02)	< 0.001	0.234	183 (17.17)	554 (17.32)	0.91	0.004
	Female	940 (83.48)	46,721 (73.98)			883 (82.83)	2644 (82.68)		
Operation year	2012	204 (18.12)	10,371 (16.41)	0.368	0.045	190 (17.82)	555 (17.53)	0.65	0.008
	2013	346 (30.73)	19,379 (30.66)		0.001	329 (30.86)	1000 (31.27)		0.009
	2014	337 (29.93)	19,068 (30.16)		0.005	323 (30.30)	921 (28.8)		0.033
	2015	239 (21.23)	14,397 (22.77)		0.037	224 (21.01)	722 (22.58)		0.038
Comorbidity	Hypertension	464 (41.21)	37,550 (59.40)	< 0.001	0.37	462 (43.34)	1403 (43.87)	0.762	0.011
	Diabetes mellitus	150 (13.32)	16,035 (25.37)	< 0.001	0.309	149 (13.98)	461 (14.42)	0.724	0.013
	Hyperlipidemia	148 (13.14)	16,093 (25.46)	< 0.001	0.316	148 (13.88)	454 (14.20)	0.8	0.009
	COPD	101 (8.97)	4604 (7.28)	0.031	0.062	97 (9.1)	270 (8.44)	0.508	0.023
	UTI	24 (2.13)	2088 (3.30)	0.029	0.072	23 (2.16)	74 (2.31)	0.767	0.01
	CHF	24 (2.13)	1761 (2.79)	0.185	0.043	23 (2.16)	66 (2.06)	0.853	0.007
	Cancer	92 (8.17)	4724 (7.47)	0.378	0.026	87 (8.16)	267 (8.35)	0.848	0.007
	CRD	29 (2.58)	2181 (3.45)	0.11	0.051	28 (2.63)	79 (2.47)	0.778	0.01
	Peripheral vascular disease	6 (0.53)	518 (0.82)	0.289	0.035	6 (0.56)	22 (0.69)	0.662	0.017
	Anemia	67 (5.95)	909 (1.44)	< 0.001	0.241	27 (2.53)	141 (4.41)	0.006	0.103
	Valvular heart disease	22 (1.95)	1676 (2.65)	0.148	0.047	22 (2.06)	55 (1.72)	0.465	0.025
	Ischemic heart disease	79 (7.02)	8957 (14.17)	< 0.001	0.234	78 (7.32)	227 (7.1)	0.81	0.009
Hospital level	Medical center	486 (45.89)	18,256 (33.09)	< 0.001		450 (44.91)	956 (33.17)	< 0.001	
	Regional hospital	350 (33.05)	20,496 (37.14)			338 (33.73)	1053 (36.54)		
	Local hospital	223 (21.06)	16,427 (29.77)			214 (21.36)	873 (30.29)		
Length of stay (days)		6.66 ± 2.21	6.69 ± 2.38	0.611		6.67 ± 2.2	6.75 ± 2.6	0.335	
Blood transfusion		559 (49.64)	26,177 (41.41)	< 0.001		525 (49.25)	1220 (38.15)	< 0.001	
Medications	NSAIDs	1107 (98.31)	58,210 (92.08)	< 0.001		1049 (98.41)	2923 (91.4)	< 0.001	
	Steroids	901 (80.02)	12,809 (20.26)	< 0.001		849 (79.64)	642 (20.08)	< 0.001	
	DMARDs	1023 (90.85)	N/A			970 (90.99)	N/A		
	anti-TNF-α	178 (15.81)	N/A			167 (15.67)	N/A		
90-day infection		29 (2.58)	1680 (2.66)	0.865		27 (2.53)	102 (3.19)	0.278	

CI, confidence interval; CHF, congestive heart failure; COPD, chronic obstructive pulmonary disease; CRD, chronic renal disease; N/A, not applicable; NSAIDs, nonsteroidal anti-inflammatory drugs; OA, osteoarthritis; OR, odds ratio; PS, propensity score; RA, rheumatoid arthritis; SMD, standardized mean difference; UTI, urinary tract infection.

On average, the RA patients were younger than the OA patients (RA: 64.76 versus OA: 70.87, *p* < 0.001), with female predominance in both groups (RA: 83.48% versus 16.52%, *p* < 0.001; OA: 73.98% versus 26.02%, *p* < 0.001). As for comorbidities at 6 months before TKA, the OA group had a higher incidence of hypertension (59.40% versus 41.21%), diabetes mellitus (25.37% versus 13.32%), hyperlipidemia (25.46% versus 13.14%), and ischemic artery disease (14.17% versus 7.02%) than the RA group did. More patients in the RA group experienced anemia (5.95% versus 1.44%). No significant differences were found in chronic obstructive pulmonary disease, urinary tract infection, congestive heart failure, cancer, chronic renal disease, peripheral vascular disease, or valvular heart disease occurrence. The RA patients also had higher rates of blood transfusion than the OA patients (49.64% versus 41.41%, *p* < 0.001). NSAIDs were consumed the most by RA patients (98.31%), followed by DMARDs (90.85%), steroids (80.02%), anti-TNF-α (15.81%). NSAIDs were also consumed the most by OA patients, while DMARDs and anti-TNF-α were not applicable in this group. The rate of acute perioperative infection was comparable between the two groups (RA: 2.58% versus OA: 2.66%; *p* = 0.865).

After PS matching with RA:OA = 1:3 (Table 1), which was estimated based on sex, age, comorbidities, and operation year, a total of 1066 pairs were included. PS, age, sex, comorbidities, and operation year were similar. SMD values were all < 10%. Although OA patients had a higher incidence of anemia, the SMD was 10.3%. After PS matching, no significant difference in surgical site infection was noted between the two groups (RA: 2.53% versus OA: 3.19%; *p* = 0.278).

We analyzed the RA and OA patients at 1 year postoperatively. The data are shown in Table 2. The surgical site infection rate was comparable between the RA and OA groups (RA: 3.04% versus OA: 2.91%; *p* = 0.812). After PS matching with RA:OA = 1:3, no significant difference in 1-year surgical site infection was noted between the two groups (RA: 2.02% versus OA: 2.97%; *p* = 0.143). After PS weighting with SMRW and conditional logistic regression, the risk of acute infection between the two groups was found to be similar according to the two analyses and two correction methods (Table 3). We also estimated the infection risk of RA patients at 1 year after TKA. The risks of infection between the two groups were also comparable according to the two analyses and two correction methods. Other significant risk factors were also presented. For 90-day infection, after PS SMRW-weighted logistic regression analysis, risk factors were sex and blood transfusion. We found that hospital level was significantly associated with infection by PS matching analysis. For 1-year infection, hospital level was a marginal significant risk factor by PS SMRW-weighted logistic regression.

**Table 2:** Characteristics of the study population at baseline and one-year follow-up after TKA.

		Original data				After 1:3 PS matching			
		RA (n = 887)	OA (n = 48,818)	*p*-value	SMD	RA (n = 842)	OA (n = 2526)	*p*-value	SMD
PS		0.05 ± 0.06	0.02 ± 0.02	< 0.001	0.671	0.03 ± 0.03	0.03 ± 0.03	0.1	< 0.001
Age		64.93 ± 10.11	70.82 ± 8.18	< 0.001	0.641	65.75 ± 9.4	65.6 ± 9.25	0.673	0.016
Sex	Male	139 (15.67)	12,589 (25.81)	< 0.001	0.252	137 (16.27)	426 (16.86)	0.689	0.016
	Female	748 (84.33)	36,186 (74.19)			705 (83.73)	2100 (83.14)		
Operation year	2012	204 (18.12)	10,371 (16.41)	0.445	0.045	190 (22.57)	559 (22.13)	0.939	0.011
	2013	346 (30.73)	19,379 (30.66)		0.001	329 (39.07)	982 (38.88)		0.004
	2014	337 (29.93)	19,068 (30.16)		0.005	323 (38.36)	985 (38.99)		0.013
Comorbidity	Hypertension	359 (40.47)	29,015 (59.44)	< 0.001	0.386	358 (42.52)	1100 (43.55)	0.602	0.021
	Diabetes mellitus	106 (11.95)	12,269 (25.13)	< 0.001	0.344	105 (12.47)	359 (14.21)	0.204	0.051
	Hyperlipidemia	113 (12.74)	12,260 (25.11)	< 0.001	0.32	113 (13.42)	371 (14.69)	0.364	0.037
	COPD	74 (8.34)	3581 (7.34)	0.255	0.037	70 (8.31)	202 (8)	0.77	0.011
	UTI	20 (2.25)	1594 (3.27)	0.093	0.062	19 (2.26)	59 (2.34)	0.895	0.005
	CHF	20 (2.25)	1373 (2.81)	0.319	0.036	19 (2.26)	35 (1.39)	0.081	0.065
	Cancer	64 (7.22)	3582 (7.34)	0.89	0.005	62 (7.36)	163 (6.45)	0.36	0.036
	CRD	17 (1.92)	1596 (3.27)	0.024	0.085	16 (1.9)	64 (2.53)	0.296	0.043
	Peripheral vascular disease	6 (0.68)	392 (0.8)	0.675	0.014	6 (0.71)	11 (0.44)	0.397	0.036
	Anemia	50 (5.64)	692 (1.42)	< 0.001	0.23	18 (2.14)	84 (3.33)	0.082	0.073
	Valvular heart disease	18 (2.03)	1313 (2.69)	0.227	0.044	18 (2.14)	40 (1.58)	0.284	0.042
	Ischemic heart disease	61 (6.88)	6906 (14.15)	< 0.001	0.239	59 (7.01)	167 (6.61)	0.691	0.016
Hospital level	Medical center	386 (46.01)	14,262 (33.15)	< 0.001		358 (45.03)	711 (31.32)	< 0.001	
	Regional hospital	270 (32.18)	16,036 (37.27)			262 (32.96)	852 (37.53)		
	Local hospital	183 (21.81)	12,727 (29.58)			175 (22.01)	707 (31.15)		
Length of stay (days)		6.77 ± 2.27	6.76 ± 2.32	0.904		6.77 ± 2.26	6.77 ± 2.11	0.986	
Blood transfusion		448 (50.51)	20,714 (42.43)	< 0.001		422 (50.12)	991 (39.23)	< 0.001	
Medications	NSAIDs	872 (98.31)	44,957 (92.09)	< 0.001		828 (98.34)	2334 (92.4)	< 0.001	
	Steroids	707 (79.71)	9731 (19.93)	< 0.001		667 (79.22)	502 (19.87)	< 0.001	
	DMARDs	809 (91.21)	N/A			765 (90.86)	N/A		
	anti-TNF-α	138 (15.56)	N/A			132 (15.68)	N/A		
One-year infection		27 (3.04)	1420 (2.91)	0.812		17 (2.02)	75 (2.97)	0.143	

CI, confidence interval; CHF, congestive heart failure; COPD, chronic obstructive pulmonary disease; CRD, chronic renal disease; N/A, not applicable; NSAIDs, nonsteroidal anti-inflammatory drugs; OA, osteoarthritis; OR, odds ratio; PS, propensity score; RA, rheumatoid arthritis; SMD, standardized mean difference; UTI, urinary tract infection.

**Table 3:** The adjusted odds ratio of perioperative infection for patients with RA.

		90-day infection		One-year infection	
		OR (95% CI)	*p*-value	OR (95% CI)	*p*-value
**PS SMRW-weighted logistic regression**^**a**^		(RA:OA = 1126:63,215)		(RA:OA = 887:48,818)	
Model 1	OA	1	0.955	1	0.924
	RA	0.985 (0.585, 1.658)		1.027 (0.595, 1.773)	
Model 2	OA	1	0.884	1	0.838
	RA	0.96 (0.553, 1.665)		1.062 (0.598, 1.885)	
	Sex				
	Male	1			
	Female	0.496 (0.268, 0.916)	0.025*		
	Blood transfusion	1.650 (0.934, 2.914)	0.085^+^		
	Hospital level				
	Medical center			1	
	Regional hospital			1.197 (0.585, 2.447)	0.623
	Local hospital			1.805 (0.898, 3.628)	0.097^+^
**PS matching**^**b**^		(RA:OA = 1066:3198)		(RA:OA = 842:2526)	
Model 1	OA	1	1.000	1	0.613
	RA	1.000 (0.581, 1.722)		0.842 (0.433, 1.638)	
Model 2	OA	1	0.598	1	0.26
	RA	0.81 (0.371, 1.768)		0.463 (0.121, 1.766)	
	Hospital level				
	Medical center	1			
	Regional hospital	0.831 (0.135, 5.136)	0.843		
	Local hospital	5.862 (1.018, 33.765)	0.048*		

CI, confidence interval; OA, osteoarthritis; OR, odds ratio; PS, propensity score; RA, rheumatoid arthritis. Model 1: correction with diagnosis (RA/OA); Model 2: correction with diagnosis (RA/OA), sex, age, hypertension, hyperlipidemia, diabetes mellitus, length of stay, blood transfusion, and hospital level.

*p-value < 0.05; ^+^0.05 < *p*-Value < 0.1

^a^Weighted logistic regression for original data, using standardized mortality ratio weighting (SMRW).

^b^PS matching with conditional logistic regression.

## Discussion

In this population-based cohort study, we found no significant difference in acute TKA surgical site infection risk between RA and OA patients while controlling for potential confounders. When the follow-up period was extended to 1 year after TKA, the results remained similar. Previous studies showed conflicting results when comparing RA and OA patients in terms of surgical site infection. Chesney et al. enrolled 1509 RA or OA patients who had undergone TKA. The superficial and deep infection rates did not show a significant difference (*p* > 0.05) between the two groups^18^. LoVerde et al. matched RA (n = 159) with OA (n = 318) patients in a 1:2 ratio and discovered a slightly higher superficial infection rate in the OA group than that in the RA group (OA: 9.4% versus RA: 5%), with marginal significance (*p* = 0.09)^19^. The results of both studies are compatible with our result, considering the original data, according to which RA and OA patients have a similar infection rate within 90 days after TKA.

Moreover, Zhu et al. discussed the risk factors of surgical site infection after total joint replacement. RA itself and the use of immunosuppressive drugs could both increase the infection rate (*p* < 0.001)^7^. In a systemic review, Lee et al. reported that superficial postoperative infection occurred in 15 of 258 RA patients and in 77 of 1609 OA patients (5.8% versus 4.7%), and deep infection occurred in 229 of 7651 RA patients and in 642 of 68,628 OA patients (3% versus 0.9%). Only the deep infection rate showed a significant difference (*p* < 0.001)^17^. Ravi et al. also concluded that RA patients have a higher infection rate than OA patients do after TKA (1.2% versus 0.8%; odds ratio = 1.45; 95% confidence interval: 1.02–2.07)^13^. Our study results are not consistent with the results of the above studies, which may be due to the methodological differences. For example, one Danish nationwide cohort study considered prosthetic joint infection as infection that occurred at 1 year after operation^15^, while Ravi et al. defined postoperative infection as infection that occurred within 2 years^13^.

We defined acute infection as infection occurring within 90 days after the operation, as it could appropriately reflect the infection risk related to surgery. To distinguish whether anti-rheumatic drugs affect the immune system perioperatively, we re-analyzed the infection rate at 1 year after operation with the same statistical analyses. The risk of infection was similar between RA and OA patients, and this indicated that their susceptibility to infection was the same.

RA patients use three kinds of anti-rheumatic drugs to control disease activity: DMARDs, anti-TNF-α, and steroids. These drugs have been suspected to provoke infectious events^24^. Doctors choose different regimens according to patients’ disease activity and the protocol from National Health Insurance. During the perioperative period, anti-rheumatic therapy should be adjusted to reach a balance between avoidance of infection and exacerbation of RA. Unfortunately, there is still no consensus on how to attain this goal^25,26^. The adjustment of long-term anti-rheumatic therapy before and after operation as well as patients’ compliance with drug schedules may affect the immune system and influence the infection caused by either blood transfusion or operation. We conducted another retrospective study also using the NHIRD to estimate the acute TKA surgical site infection rate of RA patients using different anti-rheumatic drug regimens. The risk of SSI was not significantly different between DMARDs, corticosteroid and anti-TNF-α in RA patients (DMARDs versus corticosteroid: odds ratio = 2.73, 95% confidence interval: 0.354–21.031; anti-TNF-α versus corticosteroid: odds ratio = 2.662, 95% confidence interval: 0.25–28.342; anti-TNF-α versus DMARDs: odds ratio = 0.862, 95% confidence interval: 0.291–2.548)^27^.

RA patients tend to develop anemia because of bone marrow suppression due to chronic disease or medication use^28^. These patients are more susceptible to postoperative anemia and are more likely to require a blood transfusion. Blood transfusions may increase the risk of infection by triggering transfusion-related immunomodulation^29,30^. However, in our study, RA patients had a higher rate of anemia and a higher blood transfusion rate after operation, and infection rate seemed to be unaffected, even after PS matching.

The establishment of RA should be diagnosed according to the new ACR/EULAR RA criteria^31^. However, Gabriel reported poor agreement between the diagnostic coding for RA in a database and the diagnosis based on clinical criteria in medical records^32^. Bongartz et al. also concluded that 31% of the patients registered as having RA did not meet the ACR criteria for this diagnosis^33^. Such a high rate of misclassification may threaten our cohort study, which uses the database diagnosis as the sole source of diagnostic classification. We not only enrolled RA patients by searching for the ICD-9-CM code in the database but also collected anti-rheumatic drug use information using the Anatomical Therapeutic Chemical code to enhance the accuracy of RA diagnosis in order to diminish selection bias.

There are some limitations in this study. First, we collected data from the NHIRD, which has no detailed information on other potential confounders, including disease activity, obesity, length of surgery time, blood loss or other non-measurable factors (e.g., the condition of the knee, surgical technique, surgical approach, type of implant, etc.). Additionally, the NHIRD has no information regarding whether infections are superficial or deep. Therefore, we cannot analyze these risk factors or outcomes in this study. Second, the generally low surgical site infection rate may underpower our conclusion, and this may not be addressed by extending the accrual period and, potentially, the follow-up window. However, in the clinical setting, such a low infection rate is what clinicians expect in patients receiving TKA. Finally, we did not have complete records of the medications taken by patients and were unable to make assertions about the adequacy and adjustment of anti-rheumatic medication in the perioperative period.

## Conclusions

According to this thorough collection from the database of the whole population in Taiwan, RA patients do not have a higher risk of acute or even 1-year postoperative surgical site infection after TKA compared to OA patients. The current treatment strategy for patients with RA is safe and appropriate if they require TKA.

## Supplementary Information

Supplementary Table S1.

## Data Availability

All data generated or analyzed during this study are included in this published article.
