# Supplementary material for: Acute surgical site infection after total knee arthroplasty in patients with rheumatoid arthritis versus osteoarthritis
Source: Sci Rep. 2021 Nov 22;11:22704. doi: 10.1038/s41598-021-02153-x (PMC8609034; doi:10.1038/s41598-021-02153-x)
Supplement: Supplementary file 1 — Supplementary Table S1. [file 41598_2021_2153_MOESM1_ESM.pdf]

Appendix A

Table A1. ICD-9-CM codes for diseases and orders. ICD-9-CM: International Classification of Diseases, Ninth Revision, Clinical Modification.

	**Diagnosis**	**ICD-9-CM Code**
Disease	Osteoarthritis	715.0
	Rheumatoid arthritis	714.0
Comorbidity	Hypertension	401–405
	Diabetes mellitus	250
	Hyperlipidemia	272
	Chronic obstructive pulmonary disease	490–496
	Urinary tract infection	599.0
	Congestive heart failure	428
	Cancer	140–239
	Chronic renal disease	585
	Peripheral vascular disease	443.9
	Anemia	280–285
	Valvular heart disease	394–396, 424, 746
	Ischemic heart disease	410–414
Postoperative infection	Infection and inflammatory reaction due to unspecified internal prosthetic device, implant, and graft	996.60
	Infection and inflammatory reaction due to internal joint prosthesis	996.66
	Infection and inflammatory reaction due to other internal orthopedic device, implant, and graft	996.67
	Infection and inflammatory reaction due to other internal prosthetic device, implant, and graft	996.69
	Disruption of operation wound	998.3
	Postoperative infection	998.5
	Other postoperative infection	998.59
	Pyogenic arthritis, lower leg	711.06
	Pyogenic arthritis, other specified sites	711.08
	Unspecified osteomyelitis, lower leg	730.26
	Unspecified osteomyelitis, other specified sites	730.28
	**Order**	**Order Code**
	Debridement, <5 cm	48004C
	Debridement, 5–10 cm	48005C
	Debridement, >10 cm	48006C
	Sequestrectomy, tibia, fibula, radius, ulna, humerus, or pelvis	64053B
	Arthrotomy for acute septic shoulder, elbow, wrist, ankle	64004C
	Hip, knee, shoulder remove of prosthesis	64198B
Blood transfusion	Packed red blood cells	93001C
	Washed red blood cells	93002C
	Leucocyte-poor red blood cells	93019C
	Procedure	ICD-9-CM procedure code
	Total knee arthroplasty	81.54
